# A Cross Sectional Study of the Prevalence of Preputial and Penile Scrotal Abnormalities among Clients Undergoing Voluntary Medical Male Circumcision in Soweto, South Africa

**DOI:** 10.1371/journal.pone.0156265

**Published:** 2016-06-02

**Authors:** Hillary Mukudu, Kennedy Otwombe, Fatima Laher, Erica Lazarus, Mmatsie Manentsa, Limakatso Lebina, Victor Mapulanga, Kasonde Bowa, Neil Martinson

**Affiliations:** 1 Perinatal HIV Research Unit (PHRU), MRC Soweto Matlosana Collaborating Centre for HIV/AIDS and TB, Faculty of Health Sciences, University of the Witwatersrand, Johannesburg, South Africa; 2 School of Medicine, University of Zambia, Lusaka, Zambia; 3 School of Medicine, Copperbelt University, Ndola, Zambia; University of Pittsburgh, UNITED STATES

## Abstract

**Objective:**

Medical device use is currently approved for males without preputial or major penile scrotal abnormalities for voluntary medical male circumcision (VMMC). We determined the prevalence of preputial abnormalities at a busy VMMC centre in Soweto, South Africa.

**Methods:**

This was a cross-sectional record review at a high-volume VMMC centre in South Africa. We collated pre-circumcision demographic and genital examination findings from clients 8 years and older who had undergone VMMC from 01 May 2013 to 30 April 2014. Logistic regression was used to determine factors associated with preputial abnormalities.

**Findings:**

During the review period, 6861 circumcisions were conducted and 37.1% (n = 2543) were 8–13 year olds. Median age was 15 years (IQR: 12–23 years). Fifteen percent (n = 1030) had preputial abnormalities or major penile scrotal abnormalities. Age-specific prevalence of preputial or major genital abnormalities were 27.3%, 10.6% and 6.0% in 8–13, 14–18 and > 18 year olds respectively. The odds of preputial or major penile scrotal abnormality were higher in younger clients aged 8–13 years (OR = 5.9; 95% CI = 4.8–7.1) and 14–18 years (OR = 1.9; 95% CI = 1.5–2.4) compared to older clients above18 years and in those testing for HIV outside our clinic network (OR = 1.9; 95% CI = 1.4–2.7).

**Conclusion:**

The high prevalence of preputial and penile scrotal abnormalities observed suggests a need for VMMC sites to provide for both open surgical and devices methods in the provision of VMMC services. This is especially so among young male subjects presenting themselves for VMMC services at the various sites being developed in sub Saharan African countries.

## Introduction

Voluntary medical male circumcision (VMMC) was recommended as an HIV prevention strategy in 2007, and settings with high HIV incidence and low circumcision rate have specifically been emphasised as a priority for such programmes.[1] By 2014, more than nine million circumcisions had been performed in these countries, of which almost two million were done in South Africa.[2] The roll-out of VMMC provides males with an entry point into the health system,[3] a critical aspect because males typically have less health-seeking behaviour than females.[4],[5]

Three open surgical (scalpel-based) methods using local anaesthesia were recommended for VMMC by the World Health Organisation (WHO) and three medical devices in neonates and infants.[3] However, in order to meet the increasing demand for VMMC in adults, two adult medical devices were recommended by WHO in 2013[1]. The WHO approved two foreskin crushing devices, PrePex^®^ and ShangRing^®^, following clinical trials conducted in adults in Zimbabwe, Rwanda, Zambia and Kenya.[6],[7],[8],[9],[10],[11],[12],[13],[14] These clinical trials on PrePex^®^ and ShangRing^®^ excluded males with preputial or penile scrotal abnormalities and disease, such as: active genital infection, phimosis, paraphimosis, warts or ulceration of the prepuce, torn or tight frenulum, hypospadias and any other penile diseases, anatomical abnormalities or other condition that, in the investigators’ opinion, would make device placement difficult for the VMMC procedure. The WHO technical committee that evaluated PrePex^®^ and ShangRing^®^ found that 7% and 1% of trial volunteers respectively were ineligible for circumcision.[6].

In South Africa, 16.7% of 15–24 year old males reported sexual debut before age 15. [15] VMMC for younger males may therefore be appropriate, especially considering its protective effect against HIV acquisition is durable.[16] However, WHO guidelines restrict use of medical devices to males 18 years and above, with recommendations for younger males to be considered after more evidence is collected relating to those younger than 18 years of age.[17] As a result in 2015, WHO included age group 13–18 years to be done in the context of active surveillance for the PrePex^®^ device.[18] The ShangRing^®^ has also been pre-qualified for minimum age of 13 years.[19]Although there are reports showing that the incidence and prevalence of phimosis is higher in younger males,[20],[21],[22] none of these studies were conducted in countries with high HIV prevalence and low circumcision rate where VMMC roll out is recommended. To our knowledge, this is the first study to determine the prevalence of preputial and penile scrotal abnormalities among boys between 8 and 18 years of age who present for VMMC.

## Methods

### Setting and study design

We conducted a review of medical records of clients circumcised at Khula Ndoda Medical Male Circumcision programme (KNMMC). Established in 2010, KNMMC is located in one of the largest hospitals in the world, the Chris Hani Baragwanath Academic Hospital in Soweto, South Africa. For clients younger than 18 years of age, parents/guardians/care-givers must provide written consent with verbal client assent for circumcision and/or HIV testing. Consent documents are provided in eleven languages. However, for study procedures, since consent could not be provided by the participants the data was anonymised and de-identified prior to analysis. Between 2010 and 2014, an estimated 30,000 males were circumcised at KNMMC. Prior to circumcision, KNMMC offers free HIV counselling and testing for all clients who do not have an HIV test result which could have been done at any health centre at least three months before the date of circumcision (confirmed by production of the HIV test result from the testing centre), and those who are HIV infected and eligible for treatment are first referred for treatment prior to circumcision. Circumcision for those eligible for antiretroviral therapy was done only when a repeat CD4 count after a minimum of six months on treatment was equal to or more than 350cells/ml. After an HIV test is offered, a professional nurse, clinical associate or doctor screens for medical or surgical conditions. The medical records maintain these data, together with demographic, procedural, outcome and follow-up notes.

During the period under review, KNMMC offered surgical circumcision. The forceps-guided method was primarily used, during which the aperture of the foreskin is dilated and adhesions of the foreskin to the glans penis are separated. However, the dorsal slit method was also used in some circumstances, for example for children less than 15 years, for those with phimosis, or for any other condition that precludes the use of the forceps-guided method.

### Data collation

All records for clients 8 years and older who were circumcised during the one year period from 01 May 2013 to 30 April 2014 were anonymised and de-identified prior to analysis. For those < 18 years of age, written assent was obtained from the child and consent from the parent/guardian while those aged 18 years or older provided written informed consent. Consent was obtained for both HIV testing and circumcision and was recorded on a consent form. It allowed for their clinical records to be used in this study. This approach was approved by University of the Witwatersrand Human Research Ethics Committee. None of the co-authors had access to the participant files or identifying information. We collated the following variables from the screening visit: age, HIV test result, HIV testing site, and examination findings. Data were verified through double entry. Epi Info 6 software version 3.5.4 (Centre for Disease Control and Prevention, Atlanta, Georgia 30333, USA) was used.

### Definition of Variables

Age was categorised into 8–13, 14–18 and >18 years based on data from Sub-Saharan Africa on Tanner staging in males undergoing circumcision.[23]

HIV testing sites were categorised as “KNMMC” (i.e. conducted by KNMMC staff either at the circumcision site or at mobile caravan HIV testing facilities in the community), and “Non-KNMMC” (i.e. conducted at other clinics, and results had to be less than three months old at the time of circumcision).

The HIV test results were categorised as negative, positive, and refused/not tested. The latter category included those ineligible to give consent i.e. children below 12 years of age[24] whose parent or guardian had consented to circumcision but had not signed consent for HIV testing.

A professional nurse, clinical associate or medical doctor noted male genital abnormalities in the examination findings. The diagnosis of these abnormalities was based on clinic guidelines and the clinical expertise of the attending health professional and when necessary, confirmed by a general surgeon or urologist. For the purposes of this study, a preputial abnormality was defined as any abnormality congenital or acquired on the foreskin preventing full protraction (complete covering of the glans) or full retraction of the foreskin (exposure of the glans and the full length of the coronal sulcus). Penile scrotal abnormality was defined as any congenital or acquired anomaly deemed by the VMMC provider to require urological correction, evaluation or treatment.

### Statistical Analysis

Participants were categorised into the age groups 8–13, 14–18 and >18 years and their median ages and interquartile ranges determined. No further age categories were explored above 18 years. Frequencies and their percentages for HIV testing sites, HIV test results, and abnormal genital findings were determined overall and by age group. For the purpose of logistic regression modelling, those with genital abnormalities were classified as 1 while the rest were classified as 0. Factors associated with abnormal genital findings were determined by univariate and multivariate logistic regressions. All variables with a significant p-value at the univariate analysis were considered for entry into the multivariate. Goodness of fit of the modelling was assessed by the Hosmer-Lemeshow test. All statistical analysis were conducted using SAS Enterprise Guide 5.1.

### Ethical Considerations

The study was approved by the University of the Witwatersrand Human Research Ethics Committee.

## Results

For the period under review, 6861 client records were identified and their characteristics are shown in Table 1. Of these, 2543 (37.1%), 1725 (25.1%) and 2593 (37.8%) were aged 8–13, 14–18 and > 18 years respectively. Overall, the median age was 15 years (IQR: 12–23) and the age range was 8 to 78 years. The median age for 8–13, 14–18 and > 18 year olds, was 12 (IQR: 11–13), 15 (14–17) and 26 (IQR: 22–31) respectively.

**Table 1:** Characteristics of clients who presented for VMMC by age in Soweto.

Variable	Total	8–13 years	14–18 years	>18 years
**N**	6861	2543 (37.1%)	1725 (25.1)	2593 (37.8%)
**Median age, years (IQR)**	15 (12–23)	12 (11–13)	15 (14–17)	26 (22–31)
HIV testing site*, n, (%)				
***KNMMC***	6292 (91.7%)	2153 (84.7%)	1689 (97.9%)	2450 (94.5%)
***Non-KNMMC***	257 (3.8%)	124 (4.9%)	30 (1.7%)	103 (4.0%)
**HIV test result, n, (%)**				
***Negative***	6242 (91.0%)	2098 (82.5%)	1706 (98.9%)	2438 (94.0%)
***Positive***	167 (2.4%)	21 (0.8%)	16 (0.9%)	130 (5.0%)
***Refused/not tested***	447 (6.5%)	420 (16.5%)	2 (0.1%)	25 (1.0%)
**Genital abnormality, n, (%)**	1030 (15.0%)	693 (27.3%)	182 (10.6%)	155 (6.0%)
*Phimosis*	667 (64.8%)	448 (64.6%)	140 (76.9%)	79 (51.0%)
*Complete Coronal Adhesions*	185 (18.0%)	168 (24.2%)	13 (7.1%)	4 (0.2%)
*Partial Coronal Adhesions*	70 (6.8%)	56 (8.1%)	12 (6.6%)	2 (1.3%)
*Ulcer/warts*	52 (5.0%)	1 (0.1%)	6 (3.3%)	45 (29.0%)
*Undescended Testis*	13 (1.3%)	7 (1.0%)	4 (2.2%)	2 (1.3%)
*Balanitis*	12 (1.2%)	0 (0.0%)	2 (1.1%)	10 (6.5%)
*Paraphimosis*	9 (0.9%)	4 (0.6%)	2 (1.1%)	3 (1.9%)
*Penile Deformity*	5 (0.5%)	1 (0.1%)	0 (0.0%)	4 (2.6%)
*Hypospadias*	4 (0.4%)	3 (0.4%)	0 (0.0%)	1 (0.6%)
*Inguinal Hernia*	2 (0.2%)	0 (0.0%)	1 (0.5%)	1 (0.6%)
*Bruising*	2 (0.2%)	0 (0.0%)	1 (0.5%)	1 (0.6%)
*Epispadias*	1 (0.1%)	0 (0.0%)	0 (0.0%)	1 (0.6%)
*Hydrocele*	1 (0.1%)	0 (0.0%)	0 (0.0%)	1 (0.6%)
*Other*	7 (0.7%)	5 (0.7%)	1 (0.5%)	1 (0.6%)

*Some data missing therefore does not add to total; percentages calculated using available data.

About 92% of clients were tested for HIV through the KNMMC in-service testing.

Overall, 91.0% tested HIV negative while 2.4% tested positive while the rest either refused or never tested. Majority (n = 130, 5%) of those testing HIV positive were > 18 years. About 6.5% (n = 447) were untested for HIV.

A total of 1030 (15.0%) males had preputial or penile scrotal abnormalities (PPSA) of whom 693/1030 (67.3%) were 8–13 years old. The prevalence of PPSA was 27.3% in 8–13 year olds, 10.6% in 14–18 year olds and 6.0% in those >18 years.

**Fig 1:**
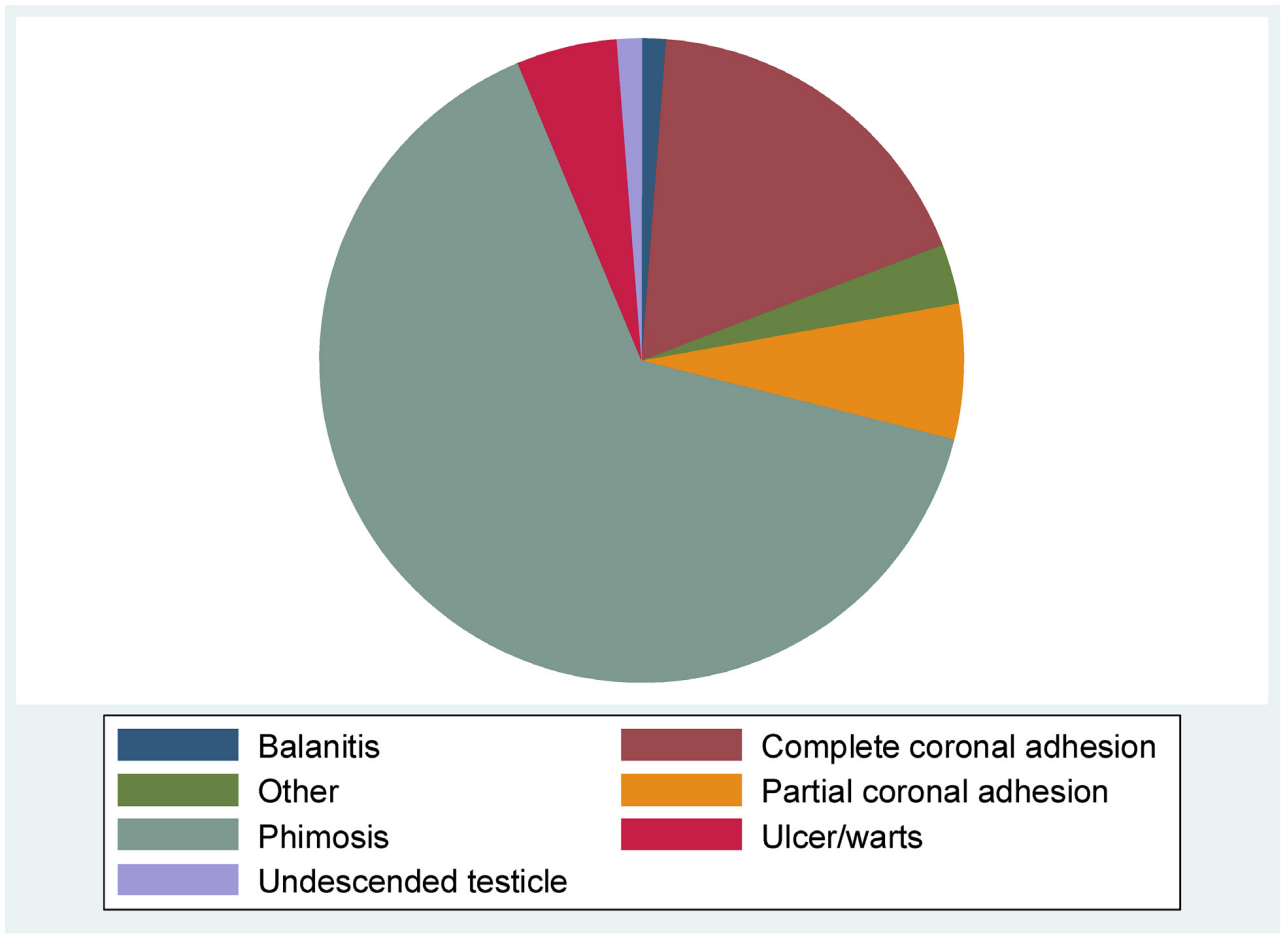
Overall distribution of genital abnormalities.

**Fig 2:**
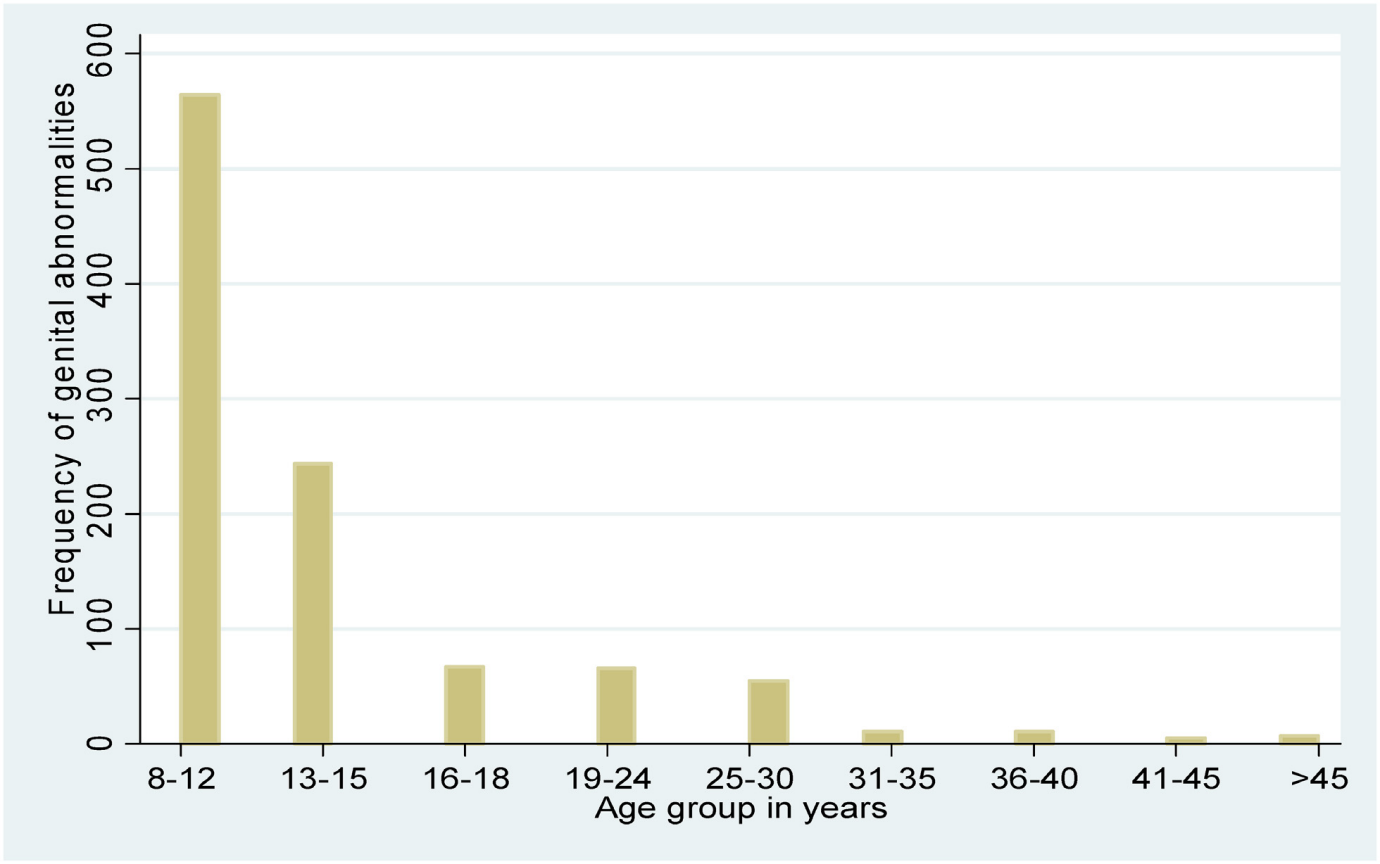
Overall distribution of genital abnormalities by age.

**Fig 3:**
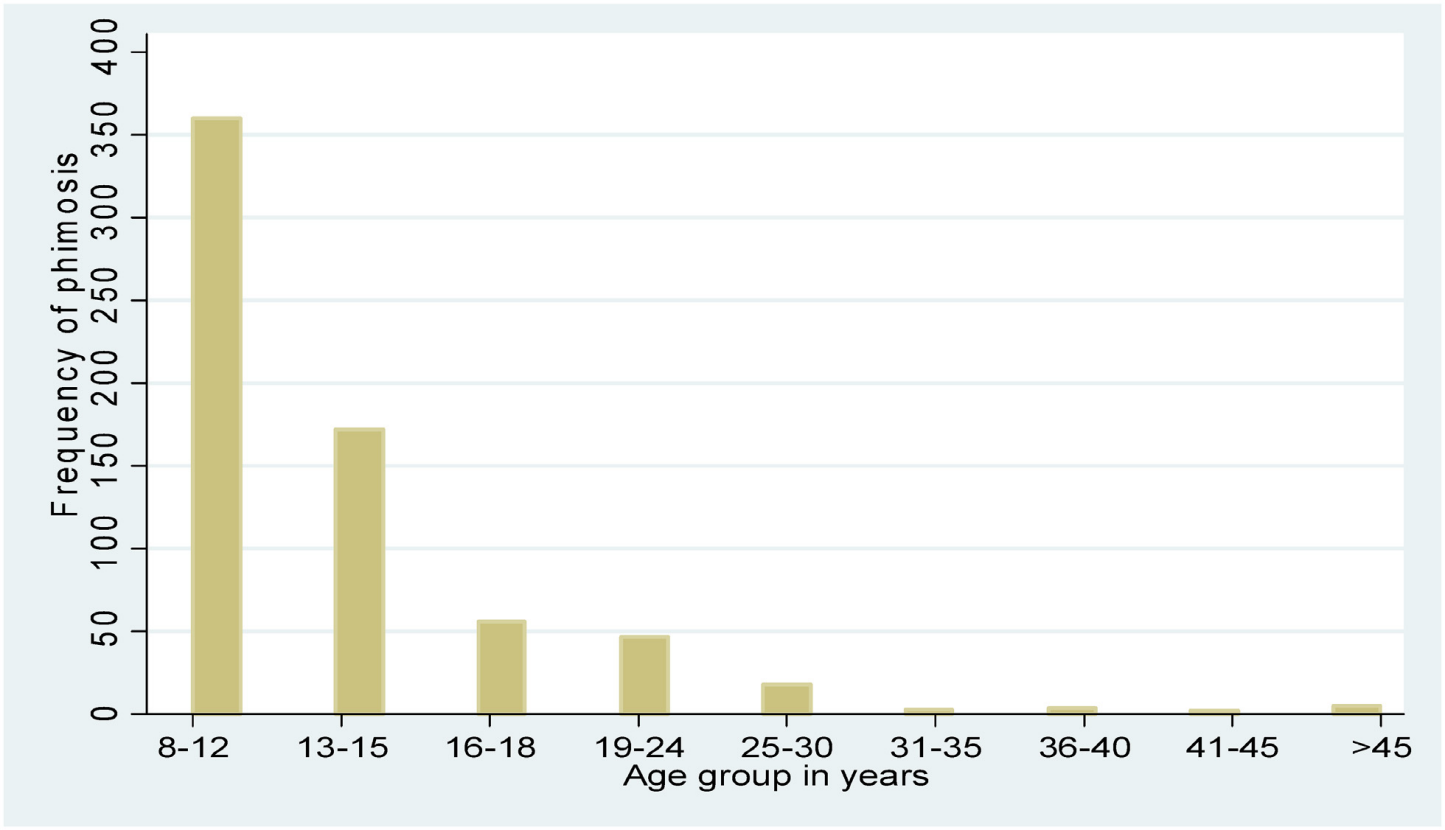
Distribution of phimosis by age.

**Fig 4:**
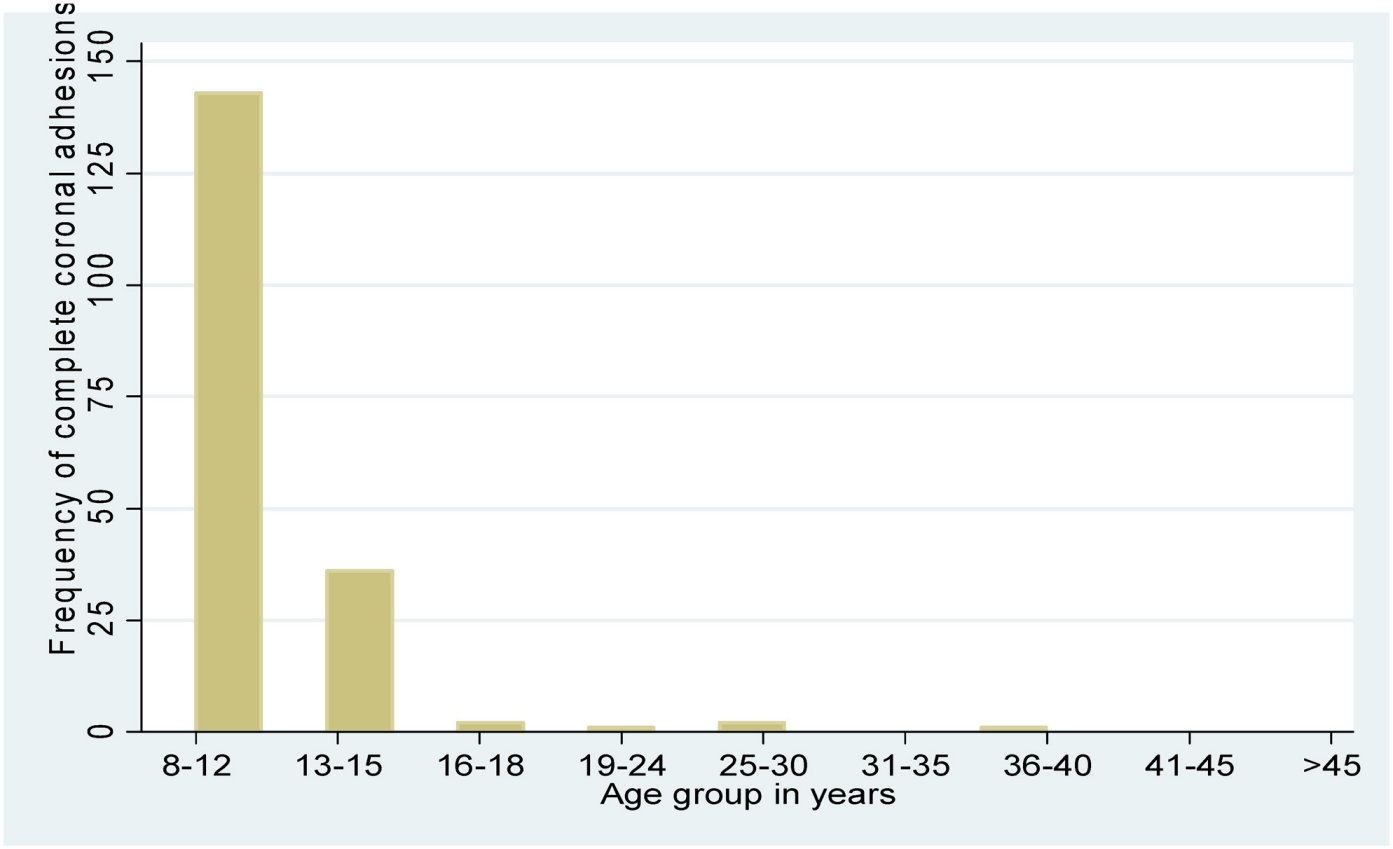
Distribution of complete coronal adhesions by age.

Figs 1 and 2 present the overall distribution of PPSA and by age group. Similarly, Figs 3 and 4 present the distribution of phimosis and complete coronal adhesions respectively. The most common PPSA were phimosis (64.8%), complete coronal adhesions (18.0%), and partial coronal adhesions (6.8%). However genital ulcers and warts were more prevalent in > 18 year olds (29%).

Table 2 shows the factors associated with PPSA. Univariate analysis identified that those who were 8–13 (OR = 5.9; 95% CI = 4.9–7.1) and 14–18 years old (OR = 1.9; 95% CI = 1.5–2.3), non-KNMMC HIV testing sites (OR = 2.2; 95% CI = 1.6–2.9) and those who refused or were untested for HIV (OR = 2.4; 95% CI = 1.9–2.9) had a higher odds of PPSA. In the multivariate analysis, age between 8–13 years (OR = 5.9; 95% CI = 4.8–7.1) and 14–18 years (OR = 1.9; 95% CI = 1.5–2.4) and non-KNMMC testing sites (OR = 1.9; 95% CI = 1.4–2.7) had higher odds of association with PPSA.

**Table 2:** Factors associated with genital abnormality in Soweto.

	Univariate		Multivariate	
Variable	OR (CI)	p-value	OR (CI)	p-value
**Age (years)**				
8–13 vs. >18	5.9 (4.9–7.1)	<0.0001	5.9 (4.8–7.1)	**<0.0001**
14–18 vs. > 18	1.9 (1.5–2.3)	<0.0001	1.9 (1.5–2.4)	**<0.0001**
**Testing site**				
Non-KNMMC vs. KNMMC	2.2 (1.6–2.9)	<0.0001	1.9 (1.4–2.7)	**<0.0001**
**Test results**				
Positive vs. Negative	0.9 (0.5–1.4)	0.56	1.04 (0.6–1.9)	0.60
Refused/Not tested vs. Negative	2.4 (1.9–2.9)	<0.0001	0.9 (0.6–1.3)	0.51

## Discussion

In this study conducted at a South African VMMC centre, the overall prevalence of PPSA was 15.0%. Our study responds to a WHO requirement for more data and we therefore document the prevalence of male PPSA that would influence access to VMMC service both using open surgery and medical devices in younger males. Young males below 13 years of age are the ideal target group for HIV prevention before the first sexual debut as advocated in WHO prevention strategies[25],[26].

The prevalence we report is more than double that reported in clinical trials in Kenya, Rwanda and Zimbabwe.[6] However, those trials excluded males below 18 years of age. Moreover, our study can account for the high overall prevalence because it was found that males below 18 years of age were about four times more likely to have PPSA. This finding has implications for the roll-out of VMMC. Our study suggests that a large proportion of pre-teen boys may not be eligible for current open surgical or medical device methods.

As expected, younger and older males had different patterns of PPSA. The proportion of males 18 years and older with ulcers and warts was higher compared to those below 18 years; likely explained by sexually transmitted infections in adults. This study also confirms findings from other countries that prevalence of phimosis is much higher in younger boys and progressively reduces with age.[9],[10],[11] This finding may help inform the design of newer medical methods for younger males with PPSA.

Our study shows that those who tested at non-KNMMC sites had twice the odds of PPSA compared to those who tested in KNMMC. This may be because clients with PPSA were referred by other health facilities to our site preferentially because it is located in a tertiary hospital. Also KNMMC is next to the urology department and receives their referred clients.

Males below 18 years old made up the largest proportion of those presenting for VMMC. This may imply that, in this community, there is demand by parents of the procedure for children. It also implies that VMMC services must plan for circumcising children.

Our findings show that one in twenty males attending VMMC were HIV-infected. This suggests that if the program is successful in attracting more men aged above 18 years, VMMC can be a significant entry point for identifying HIV positive men and linking them to care and treatment.

Our study has limitations; this is not a population-based sample but is restricted to males presenting for circumcision in Soweto. Furthermore, we likely underestimated the prevalence of PPSA as we only included those boys and men who underwent circumcision at the KNMMC clinic. There may have been others who presented directly to the hospital urology department or were referred by the KNMMC clinic to the urology department because the surgery required was considered beyond the scope of an out-patient facility. Finally, different categories of health professionals diagnosed PPSA and there may not have been diagnostic standardisation.

## Conclusion

More children than adults access VMMC, and children have a higher prevalence of PPSA. Our findings highlight the need for planning VMMC services with children in mind. Additional research is required to address VMMC in young pre-teen boys with PPSA otherwise a significant population will be unreachable by this intervention.
